# The physiological and pathological mechanisms of early embryonic development

**DOI:** 10.1016/j.fmre.2022.08.011

**Published:** 2022-08-28

**Authors:** Jian Mu, Zhou Zhou, Qing Sang, Lei Wang

**Affiliations:** a The State Key Laboratory of Genetic Engineering, Institute of Pediatrics, Children's Hospital of Fudan University, The Institutes of Biomedical Sciences, Fudan University, Shanghai 200032, China; b NHC Key Lab of Reproduction Regulation, Shanghai Institute for Biomedical and Pharmaceutical Technologies, Shanghai 200032, China

**Keywords:** Early embryonic development, Physiology, Pathology, Molecular mechanism, Pathogenesis

## Abstract

Early embryonic development is a complex process. The zygote undergoes several rounds of division to form a blastocyst, and during this process, the zygote undergoes the maternal-to-zygotic transition to gain control of embryonic development and makes two cell fate decisions to differentiate into an embryonic and two extra-embryonic lineages. With the use of new molecular biotechnologies and animal models, we can now further study the molecular mechanisms of early embryonic development and the pathological causes of early embryonic arrest. Here, we first summarize the known molecular regulatory mechanisms of early embryonic development in mice. Then we discuss the pathological factors leading to the early embryonic arrest. We hope that this review will give researchers a relatively complete view of the physiology and pathology of early embryonic development.

## Graphical Abstract

**Figure:**
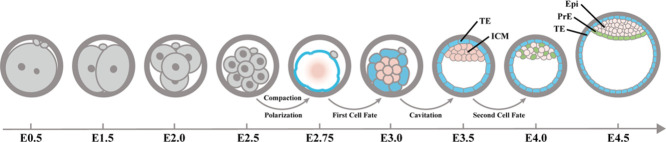

## 1 Introduction

Scientific research on embryos has been ongoing for millennia. Aristotle is considered the “first” developmental biologist, and his book *Generation of Animals* was the first to talk about embryonic development. In the 19th century, Karl Ernst von Baer developed modern embryology through his discovery of the blastula stage of development and the notochord and became the first person to observe a human egg [1]. In 1958, Mclaren and Biggers were the first to culture mouse embryos *in vitro* and subsequently produce a healthy live birth. In 1962, the *in vitro* culture of mouse embryos from the 1-cell stage was achieved, and the world's first human baby via *in vitro* fertilization (IVF) was born in 1978.

*In vitro* culture of embryos makes it easier to observe early embryonic development and to collect samples. Benefiting from advances in molecular biology techniques, scientists can observe embryonic development in more detail and study the molecular mechanisms of embryonic development from different angles. The understanding of early embryonic development has advanced tremendously in recent years.

The zygote is the beginning of life. After fertilization, the zygote begins the process early embryonic development in which it undergoes several rounds of cleavage to increase cell numbers and then forms a blastocyst, while at the same time the embryo gets rid of maternal and paternal influences to start its own life with its own genetic material. Early embryonic development is very important for new life and is thus conserved in different mammalian species, though different species differ in the details.

Early embryonic development is a precisely controlled process, and any interference involving key reactions or signaling pathways will result in low-quality embryos showing early embryonic arrest or increased embryo fragmentation. Animal models, especially transgenic mice, have helped to understand the pathological factors, including genetic factors, of early embryonic arrest, and many transgenic mouse models of early embryonic developmental arrest have been reported in studies and indexed in databases. Some genes are also reported to correspond to human early embryonic developmental arrest, and these models also help us to better understand the regulatory mechanism of early embryonic development.

In this review, we talk about early embryonic stages from zygote to blastocyst and mainly focus on the known physiological regulation of early embryonic development and the pathological factors leading to early embryonic arrest in mice.

## 2 Physiological regulation of early embryonic development

Early embryonic development must complete two processes. The first is to complete the maternal-to-zygotic transition (MZT) in which the zygote eliminates the maternal influence and ensures that further embryonic development is controlled by the zygote genome. The second process is to make two consecutive cell fate choices. After these two fate choices, the embryo differentiates into three lineages, including the epiblast (Epi), which will develop into embryonic tissues, and the trophectoderm (TE) and primitive endoderm (PrE) that will develop into extra-embryonic tissues. The two processes are regulated by complex molecular networks, and in this section we will review the known molecular mechanism of early embryonic development.

### 2.1 MZT

The fertilized oocyte has just undergone two rounds of meiosis, and immediately after fertilization it begins to undergo mitosis as a zygote. The cytoplasm of the zygote thus comes completely from the maternal oocyte, and thus the zygote needs to undergo the process of MZT to eliminate maternal factors and begin following its own genetic programming.

**Fig. 1:**
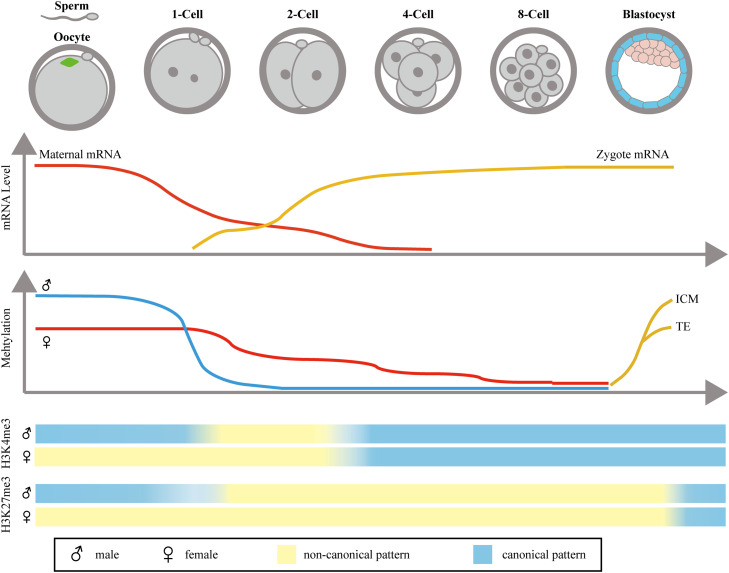
**Overview of the MZT in mice.** Maternal mRNAs are degraded after fertilization and are almost completely eliminated at the 4-cell stage. The zygote starts to transcribe a little bit at the 1-cell stage in the minor ZGA wave and then transcribes heavily at the 2-cell stage in the major ZGA wave. Paternal DNA undergoes a quick and positive demethylation, while maternal DNA undergoes passive demethylation along with DNA replication. At the early blastocyst stage, the zygote begins re-methylation in both alleles in the DNA. The oocyte has a non-canonical pattern of H3K4me3 and H3K27me3. After fertilization, the paternal genome will be erased due to histone replacement and then rebuilt with weakened levels of ncH3K4me3 and ncH3k27me3. H3K4me3 is found in both the maternal and paternal genome at the late 2-cell stage, whereas H3K27me3 is found in the late blastocyst.

The MZT mainly consists of two events, namely maternal mRNA decay and zygotic genome activation (ZGA) (Fig. 1). The aim of maternal mRNA decay is to remove the maternal mRNAs so as to eliminate their effect on embryonic development, while ZGA activates the zygote genome to begin its own transcription and translation [2]. The MZT is regulated by several factors, including epigenetic reprogramming, the subcortical maternal complex (SCMC), etc. The MZT is the first and most essential process that rebuilds the zygote's genome and cytoplasmic constituents and thus has significant influence on subsequent embryonic development.

#### 2.1.1 Maternal effect genes

Large amounts of transcripts and proteins are produced and stored in oocytes, and prior to ZGA the embryo is dependent on these maternal factors. Maternal effect genes (MEGs) are a class of genes that are transcribed in oocytes and are essential for oocyte maturation, fertilization, and early embryonic development. *Mater* (or *Nlrp5*) was the first reported mammalian MEG, discovered in 2000, and since then about 80 genes have been reported to be MEGs [3]. All of these genes are important for female fertility as confirmed in humans or in mouse models.

The SCMC is a multiprotein complex that consists of several MEGs. It is uniquely expressed in mammalian oocytes and early embryos and is important for embryonic development. It has been identified in many species, including sheep, pigs, and monkeys, and ranges in size from about 669 to 2000 kDa. As its name implies, the SCMC is located under the membrane of the oocyte [4]. The members of this complex that have been identified include *Ooep, Khdc3, Padi6, Nlrp5, Tle6, Nlrp2*, and *Zbed3*. These proteins are all maternal proteins and are expressed at the early GV stage. SCMC mRNAs are highly expressed in oocyte and early embryo, and gradually degraded during oocyte maturation and early embryonic development, whereas the proteins are stable until the early blastocyst stage [5].

Even though the SCMC is expressed and forms a complex, it does not seem to affect oocyte maturation; however, studies in knockout mice showed that it is essential for early embryonic development. *Ooep, Nlrp5, Tle6*, and *Padi6* female knockout mice are infertile because of arrest at the 2-cell stage, and *Khdc3, Nlrp2*, and *Zbed3* female knockout mice are subfertile due to impaired preimplantation embryonic development [5]. Both female and male mice with defects in these genes are healthy, while only the females are infertile. These mouse models indicate that all SCMC members have specific functions in the embryo.

The SCMC has two main functions in the embryo. The first function is to control spindle position. TLE6 interacting with cofilin, which is a key regulator of F-actin assembly, influences the F-actin network that controls symmetric mitotic division, and *Tle6* defects result in different cell sizes in the 2-cell embryo [6]. The second function involves maternal mRNA storage and decay. Cytoplasmic lattices (CPLs) are a special structure in the oocyte and the early embryo, and CPLs are considered a place to store maternal mRNAs and might be involved in protein synthesis. OOEP, NLRP5, and PADI6 co-localize at the CPLs and are required for their formation, and oocytes that lack these proteins also lack CPLs. Also, embryos lacking CPLs experience disrupted ZGA and protein synthesis. The SCMC may also be involved in the epigenetic reprogramming of the embryo, and mutations in *NLRP5* and *KHDC3L* have been associated with improper imprinting in humans [7]. With embryo cleavage, the SCMC remains in the apical cortex until eventually it is only found in the TE, and thus it appears to regulate cell fate.

Besides the SCMC, several other MEGs also regulate embryonic development prior to implantation. BTG4 is a MEG that mediates maternal mRNA decay [8], and its deficiency results in embryonic arrest in female mice and humans [9,10]. NPM2 is a histone chaperone that regulates the nucleolus in early embryonic development, and NPM2-null females are subfertile [11]. TCL1 is a MEG that is present in the embryo only until the 2-cell stage. The function of TCL1 might be involved in maternal mRNA decay, and female mice lacking *Tcl1* are subfertile [12]. Although the functions of many MEGs in embryonic development are in need of further exploration, some MEGs whose functions are better studied will be discussed later.

#### 2.1.2 Maternal mRNA decay

During oocyte maturation and the beginning of embryonic development, the oocyte/embryo is transcriptionally silent. This means that all mRNAs needed for development must be transcribed at the oocyte germinal vesicle (GV) stage. Oocytes then need to store the mRNAs and prevent them from being translated at the wrong time. Mature mRNAs are transported into the cytoplasm and their poly(A) tails are immediately shortened [13]. After deadenylation, the mRNAs have only 20–40 adenosines in their poly(A) tails and thus are silenced until needed for translation. With oocyte/embryonic development, dormant mRNAs are reactivated for translation, while large-scale maternal mRNAs are degraded [14]. The reason of mRNA decay is not only because they are used, but also because there is a programmed degradation pathway controlled. mRNA decay in the zygote is very important for further embryonic development, and over-accumulation of maternal mRNAs can affect embryonic metabolism and can result in embryonic arrest [15]. Some MEG deficiencies, such as *Padi6, Cnot6l*, and *Yap1*, disrupt this process leading to embryonic arrest [[16], [17], [18]].

Two major rounds of mRNA decay occur. The first occurs during oocyte maturation and is called M-decay. After GV breakdown, the oocyte restarts meiosis and maternal mRNAs are decayed at the same time. In the metaphase II (MII) oocyte, the abundance of mRNAs is only 25% that of the GV oocyte. M-decay starts after GV breakdown and continues to the early embryo. The second occurs during early embryonic development and is called Z-decay. In the first few divisions, the remaining maternal mRNAs are degraded at the same time that ZGA takes place. Here we mainly discuss the decay that occurs during embryonic development.

Recent studies revealed that the BTG4-CCR4-NOT(CNOT7) complex drives M-decay in oocytes and during early embryonic development [8,9,19]. The first step of degrading maternal mRNA is deadenylation because only if the maternal mRNA has a short enough poly(A) tail can DCP1/2 and the exosome degrade the mRNA [20]. The CCR4-NOT complex is involved in mRNA deadenylation [21], and it is comprised of a scaffold subunit (CNOT1), a structural subunit (CNOT2/3), catalytic subunits (CNOT6/6L and CNOT7/8), and multiple regulatory subunits, including CNOT4 and CNOT9 [22]. However, CCR4-NOT cannot function on its own and requires the BTG/Tob proteins to guide the CCR4–NOT complex to its target mRNA through interactions with CNOT7/8 [8,23]. In oocytes, the guide protein is BTG4. BTG4 interacts with CNOT7 and eIF4E, and it brings the CCR4-NOT complex into close proximity of the mRNA tail. If BTG4 is lost in the oocyte, the maternal mRNA poly(A) tails cannot be shortened [9,23].

In oocytes, BTG4 is short-lived because the SCF ubiquitin E3 ligase promptly ubiquitinates it [24]. PABPN1L is reported to protect BTG4 by blocking its binding to βTrCP1, which is a substrate adaptor of SCF in polyubiquitination [25]. PABPN1L is expressed during the MII to 2-cell stage, so BTG4 is protected at the same time in mouse oocytes. This means that M-decay will stop at the 2-cell stage, but a few maternal mRNAs will still need to be degraded. The zygote performs this job by expressing TUT4/7. At the start of ZGA the zygote expresses TEAD4, which associates with maternal YAP1 and activates the transcription of zygotic genes. TUT7 has a TEAD-binding site on its promoter, thus TUT7 is an early ZGA protein. TUT7 functions by uridylating short-tailed mRNAs, which marks them for degradation by the exosome [26]. YAP1-TEAD4-TUT7 ultimately degrades about 60% of the maternal mRNAs through Z-decay in the early embryo.

Moreover, in addition to BTG4-CCR4-NOT and YAP1-TEAD4-TUT7 the embryo has other pathways to degrade the maternal mRNAs [27]. All of these pathways synergistically degrade maternal mRNAs in the zygote and are important for zygote development. Excessive maternal mRNA accumulation can hamper zygote metabolism in various ways. For example, M-decay failure leads to ZGA failure [9], and maternal mRNA accumulation can result in superfluous maternal proteins that negatively influence zygote development.

The processes of maternal mRNA decay are similar in mice and humans, indicating that mice and humans have a conserved pathway for regulating embryonic development. The major differences are the starting point and the duration of Z-decay. Z-decay begins shortly after the start of ZGA, which occurs at the 2-cell stage in mice but at the 4 to 8-cell stage in humans [28]. The duration is also different, and Z-decay starts from the 2-cell stage and finishes at the 4-cell stage in mice, while it takes place from the 4-cell to the morula stage in humans. The human embryo thus needs more time to clear maternal mRNAs. Also, if ZGA is inhibited half of the Z-decay transcripts fail to be degraded in mouse embryos, whereas >90% fail to be degraded in human embryos.

#### 2.1.3 ZGA

The zygote is transcriptionally silent after fertilization, and ZGA is the transcriptional activation process that rebuilds and activates the genome to begin transcribing the zygote's genes. For most mammals, there are two transcription waves, an earlier minor transcription wave and a later major wave. The initiation time of ZGA is different between species, and in mice the minor ZGA occurs at the mid to late S-phase of the 1-cell stage and the major ZGA occurs at the late 2-cell stage, while in humans the major ZGA occurs at the 4 to 8-cell stage. In some species it occurs later, such as the 8 to 16-cell stage in cows [28]. Though the timing is slightly different between species, ZGA performs the same function and is important for embryonic development.

Although maternal mRNAs can interfere with detecting ZGA-activated genes, researchers can still identify the transcription profiles of ZGA through various methods. Minor ZGA and major ZGA differ not only in the timing of occurrence, but also in the amount and pattern of gene expression. Over 800 genes are activated during the minor wave in mice, and over 3500 genes are transcribed during the major wave [29]. In humans, the zygote transcribes about 1000 genes in the minor wave and about 2500 in the major wave [30]. During minor ZGA, a large part of gene and intergenic regions, and regions that code for retrotransposons have a relatively low level of transcription that result in producing non-productive transcripts without proper poly-A tailing and splicing. During major ZGA, expression of particular genes increase whereas transcription from intergenic regions decrease [31]. Both minor and major ZGA are essential to embryonic development. If the major ZGA is disturbed, the embryo would arrest at 2-cell stage in mice. Blocking minor ZGA leads to the failure of major ZGA and embryonic lethality which means minor ZGA is a prerequisite for the occurrence of major ZGA [32].

Endogenous retroviruses, LINE-1 elements, and the non-autonomous SINE elements are found to be transcribed at the beginning of ZGA and then repressed after the 2-cell stage in mice [33]. Similar to mice, the endogenous retrovirus element HERVK-HML-2 in humans is expressed at the 8-cell stage and is repressed at the blastocyst stage [34]. These elements might influence ZGA or embryonic development, and further studies are needed to determine the mechanisms behind their effects.

The mechanism of ZGA onset remains unclear. Some studies have proposed hypotheses to try to explain ZGA, and these hypotheses are not independent or mutually exclusive. Several models are discussed below, and putting them together may help us come to a better understanding of how ZGA functions in embryonic development. Here, we summarized some hypotheses that explain how to initiate the minor ZGA and induce the major ZGA.

The nucleocytoplasmic ratio model argues that the ratio of the nucleus volume to cytoplasm volume in the embryo controls the cell cycle and ZGA. For most mammals the volume of the embryo remains constant during early cleavages, and with each cleavage the volume of each embryonic cell decreases. At the same time, however, the ratio of nucleus volume to cytoplasm volume increases. Studies have shown that changing the nucleocytoplasmic ratio can influence ZGA in zebrafish and *Xenopus* [35,36]. However, although the nucleocytoplasmic ratio appears to influence embryonic development in mice, it does not seem to have an effect on ZGA.

Titration of maternal repressors is another explanatory model. It hypothesizes the existence of one or more maternally supplied repressors that repress transcription by preventing the activation of RNA polymerase II (Pol II). During early embryonic cleavages, the DNA content is doubled but the repressor content does not change. Thus continued DNA replication might weaken the repressor's power until it can no longer repress zygote transcription. Histone is thought to be one such repressor. The zygote stores a high concentration of soluble histones that bind DNA in the genome in order to prevent transcription factors (TFs) from binding to the genome [37], and studies have shown that reducing histone H3 in zebrafish and *Xenopus* embryos will promote ZGA [38,39].

The accumulation of transcription activators is a model that considers the embryo to be lacking one or more prerequisite activators prior to ZGA. After fertilization, some activators are translated and accumulated. As time passes, the embryo can start gradually transcribing its own genes through the assistance of these activators, resulting in the minor wave of transcription. Once a particular threshold is crossed, large-scale transcription can begin. TBP is thought to be one such activator. TBP is a member of the TFIID complex and helps form the Pol II pre-initiation complex, and TBP protein levels increase prior to ZGA in *Xenopus* and promote zygote transcription [40]. Some specific TFs are also involved in ZGA. In *Drosophila, Zelda* is a well-studied gene that is essential for ZGA and promotes the transcription of hundreds of genes. However, there is no *Zelda* ortholog in mammals. Some activators have been identified in mammals, including *Dux* (in mice) and *DUX4* (in humans), *YAP1, NF-YA*, and so on [28]. *DPPA2*/*DPPA4* seems to regulate transcription in “2C-like” cells but is dispensable for ZGA in 2-cell stage embryos in mice [41].

Chromatin also influences ZGA because TFs and Pol II need to access chromatin to initiate gene transcription. As noted earlier, histones repress ZGA. Moreover, histone modifications can also affect chromatin accessibility directly or indirectly through the effect of readers. H3K4me3 begins to appear prior to ZGA and is significantly enriched during ZGA to promote transcription in *Xenopus* and zebrafish [42]. Both active and inactive genes are methylated, seemingly poising embryonic genes for activation. In mice, the landscape of H3K4me3 already exists in the oocyte but is reduced in 2-cell stage embryos in order to restrict transcription [43]. Though the patterns of methylation are different in mice compared to *Xenopus* and zebrafish, they serve the same purpose in preparing for ZGA via H3K4me3 formation. H3K27ac is also thought to be related to transcriptional activation in embryonic development. H3K27ac is a widespread modification found on chromatin prior to ZGA in zebrafish, and a similar pattern is found in mice and *Drosophila*. Besides H3K4me3 and H3K27ac, some other histone modifications, such as H2K27me3 and H3K9me3, are reported to be associated with ZGA [43]. Not only histone modification, but also histones are thought to be important for ZGA. The nucleosomes of 1-cell stage embryos are composed of a unique set of histone variants. They mainly comprise H3.3 which is thought loosened chromatin structure. And H2A.X and TH2A also suggested to be involved in chromatin loosening [44].

Besides histone modification, chromatin remodeling is another pathway to regulate chromatin accessibility. Chromatin remodelers can regulate nucleosomes by promoting TFs binding to DNA. This process requires the energy from ATP, and BRG1 is the catalytic ATPase subunit of the SWI/SNF complex that remodels nucleosomes. BRG1 is a maternal gene that is essential for embryonic development in mice, and complete deficiency of BRG1 in mouse oocytes leads to reduced transcription of ∼30% of expressed genes in the embryo. Depletion of BRG1 does not affect global histone acetylation, whereas H3K4me2 levels are reduced [45].

Though there are many hypotheses about ZGA, they are not independent or mutually exclusive, and by combining these hypotheses we can get a more comprehensive view of how ZGA operates. Oocytes accumulate repressors to repress transcription and translation until required for maturation and fertilization. After fertilization the zygote undergoes cleavage, and with subsequent cleavages the repressors are diluted and some maternal mRNAs of transcription activators are translated. These activators prepare the zygote's genome for activation. At the same time, chromatin is opened up by histone modification and remodeling. All of these processes promote TFs and Pol II binding to DNA and initiating transcription. Zygote mRNAs are translated, and some newly synthesized proteins promote transcription further. This feedback quickly activates the zygote genome. Although these insights can explain the onset of ZGA in general, the detailed mechanism remains unclear. For example, it is not known how many repressors and activators exist in the zygote, what factors regulate chromatin opening, how they regulate the onset time of ZGA, and why there are different onset times between species. New research and new technologies may give answers to these and other questions.

#### 2.1.4 Epigenetic reprogramming

Mammalian somatic cells all possess the same genomic sequences, but their specific functions are dictated by the expression of specific genes at different locations and times. This regulation is ensured by DNA and histone modifications. However, the zygote results from the fusion of a differentiated oocyte and sperm, and their chromosomes are highly processed and modified. Therefore, after fertilization the zygote needs to undergo global reprogramming in order restore its totipotency, and this reprogramming involves both DNA and histone modifications. We described histone modifications above in talking about ZGA, and here we focus on global histone modifications from fertilization to the blastocyst stage. And we need to declare that although existing the divergent epigenetic reprogramming modes, the reprogramming process is largely conserved among mammals [46].

During early embryonic development, the paternal and maternal DNA undergoes different demethylation. Paternal DNA undergoes active demethylation, which occurs extremely rapidly after fertilization and prior to DNA replication. The TET family are the active DNA demethylases, and TET3 is responsible for paternal DNA demethylation in zygotes [47]. Female mice lacking TET3 are subfertile and present with a high level of DNA methylation and a low level of DNA hydroxymethylation [47]. Demethylation of the maternal genome is a passive approach relying on DNA replication. During the initial cleavages, both the paternal and maternal genomes are hypomethylated at the blastocyst stage. During this phase, the zygote's DNA will not be methylated because DNMT1 is prevented from entering the nucleus [48]. At the blastocyst stage, the embryonic DNA quickly rebuilds its *de novo* DNA methylation. The inner cell mass (ICM) and TE are methylated differently, and methylation of the ICM increases from ∼25% to ∼75% while methylation of the TE increases to ∼50% [49].

Before the first cleavage, maternal chromatin maintains its modifications. In contrast, paternal chromatin undergoes rebuilding of its histone modifications. The sperm nucleus has high concentrations of protamine, and after fertilization protamine is replaced by maternal histone that consists of the histone variant H3.3, which is essential for embryonic development [50]. Knockdown of H3.3 results in developmental arrest at the morula stage, and this phenotype can be rescued by replenishing H3.3 mRNA but not H3.1. Thus H3.3 seems to be an activated histone that is involved in transcriptional activation [51].

Histone modification of the paternal genome is quite different from that of the maternal genome after fertilization. Before the blastocyst stage, these differences are gradually eliminated. Many histone modifications are reprogrammed in early embryonic development, and here we discuss H3K4me3 and H3K27me3 in particular. H3K4me3 is a hallmark for transcription initiation, and it is usually enriched around the transcription start site [52]. However, in the oocyte the genome has a special pattern of H3K4me3 that exists as broad peaks at promoters as well as at a large number of distal loci where it is referred to as the non-canonical form of H3K4me3 (ncH3K4me3). This pattern seems to repress transcription. After the late 2-cell stage, ncH3K4me3 is removed and canonical H3K4me3 is rebuilt on the maternal genome. H3K27me3 also has a non-canonical form in the maternal genome. After fertilization, promoter-associated H3K27me3 is specifically erased from the maternal genome, whereas distal H3K27me3 remains widespread in intergenic regions, and this state is maintained until embryo implantation [53]. However, after fertilization the paternal genome undergoes a brief ncH3K4me3 modification and is then quickly rebuilt along with the maternal genome [54]. H3K27me3 of the paternal genome is lost after fertilization, but some ncH3K27me3 remains on the paternal genome [53]. When the embryo develops into the blastocyst stage at embryonic day 5.5, the H3K27me3 of the zygote genome is restored at development-related gene promoters [53]. Promoters of development genes mark bivalency at embryonic day 6.5 in the Epi to ensure rapid activation during lineage specification [55].

During early embryonic development, even though the zygotic DNA undergoes global demethylation as described above, some imprinted loci escape the wave of demethylation. Imprinting genes are essential for early embryonic development, placenta development, and implantation, and DNMT1 is needed to maintain imprinting methylation. Though most of the DNMT1 is excluded from the nucleus, a few DNMT1 molecules can enter the nucleus and specifically methylate imprinting genes [56]. DNMT1 can pass through the ZFP57-TRIM28 complex to specific locations in the imprinting control region [57], and STELLA protects zygote DNA from being demethylated [58].

Another special process in epigenetic reprogramming is X chromosome inactivation (XCI). XCI is a mechanism of dosage compensation that balances the expression levels of X-linked genes between females, who have two X chromosomes, and males, who have a single X chromosome. In most species, including humans, the inactivation of one of the X chromosomes is randomly chosen [59]. In mice, however, the paternal X chromosome is silenced in early embryonic development due to the expression of *Xist*. Paternal XCI is reversed in the ICM and is randomly selected in the Epi [60]. *Xist* is a long non-coding RNA that can regulate XCI in *cis*, and the X chromosome that expresses higher levels of *Xist* will be silenced. The mechanism of how *Xist* regulates XCI is not yet clear, but it has been suggested that H3K27me3 plays a significant role [61].

### 2.2 Early cell fate decisions

**Fig. 2:**
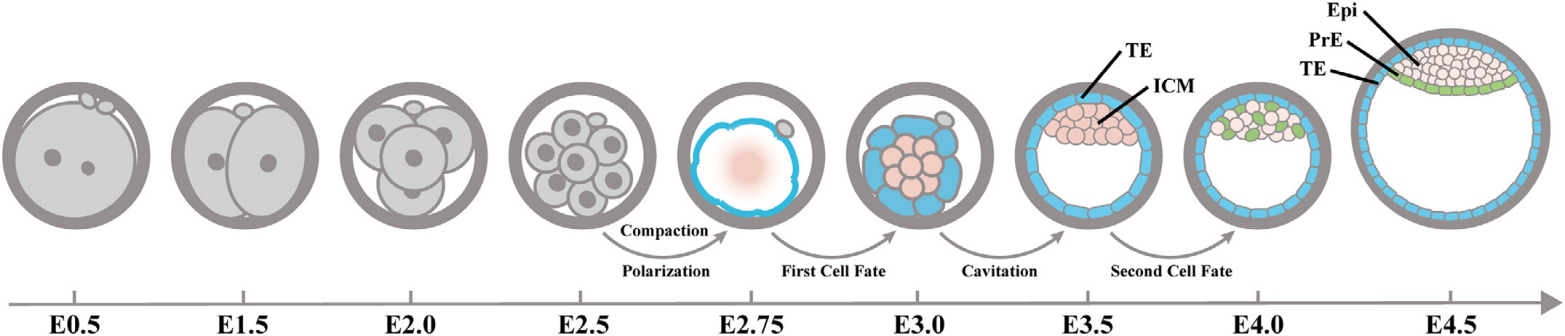
**Morphological changes over time during early embryonic development.** The zygote undergoes three divisions to reach the 8-cell stage, at which time the embryo undergoes compaction and polarization at the 8-cell stage. The blastomere then differentiates into the ICM and TE and forms a cavity. The ICM cells of the early blastocyst undergo the second fate choice, and some differentiate into the Epi and some into the PrE. PrE cells will migrate to the surface of the ICM that is exposed to the cavity. ICM: Inner Cell Mass; TE: Trophectoderm; Epi: Epiblast; PrE: Primitive Endoderm.

The MZT is essential for early embryonic development. In this process, the embryo removes the memory from the sperm and oocyte, starts its own gene transcription, and builds a new and totipotent genome. After the MZT, the zygote genome takes over genetic control and starts regulating embryonic development. At the late stage of the MZT, embryonic cells make their first fate choice. The blastomere undergoes the first differentiation, producing two cell types, the TE and the ICM. This process is divided into two stages, compaction and cavitation, based on their morphological characteristics. The choice between the TE and ICM is regulated by different molecular mechanisms. After the first fate choice, the cells of the ICM will make a second fate choice and will differentiate into the Epi or the PrE (Fig. 2). Once the blastocyst implants in the uterus the Epi will begin to develop into embryonic tissues.

#### 2.2.1 Early embryo compaction and surface polarization

After fertilization, the first three cleavages lead to reduced cell volumes. Up to the early 8-cell stage all embryonic cells have similar spherical shapes, and their surfaces have weak adhesion with each other. However, at the late 8-cell stage all of the cells undergo a process called compaction that involves increasing numbers of adherens junctions within cells. This maximizes the cells’ contact area and leads the embryo to acquire a compressed morphology, and this compaction is conserved in all mammals known so far.

The mechanism behind compaction is still not fully understood, but some factors are known to be essential for it. Extracellular calcium (Ca^2+^) influences compaction, and in Ca^2+^-free culture medium embryos do not undergo compaction. Even in embryos that are already compacted, loss of Ca^2+^ will reverse the compaction and polarization. Ca^2+^ affects compaction through epithelial cadherin (E-cad), which is a major component of adherens junctions and can be detected at the basolateral cell-to-cell contact surfaces at the 8-cell stage [62]. Interfering with E-cad function can negatively affect compaction. *Cdh1* encodes E-cad, and *Cdh1*-null zygotes can still undergo compaction due to residual maternal E-cad [63]. If the female lacks *Cdh1*, the embryo can still undergo compaction, although it will be delayed, due to zygotic E-cad expression after ZGA. Only when both the maternal genome and the zygote genome lack *Cdh1* will the embryo fail to compact at all [64]. These models indicate the importance of E-cad in compaction. E-cad is present on the entire surface of the cell membrane prior to compaction, and when compaction begins about 5 of the 8 cells extend long E-cad–dependent filopodia onto neighboring cells, thus causing all of the cells to change shape [65]. However, Maitre et al. suggested that the function of E-cad in compaction is to inhibit contractility at cell-cell contacts, and they proposed that pulsatile cell-autonomous contractility provides the force for compaction [66]. All of the proteins and factors needed for compaction already exist at the 4-cell stage, and compaction can be induced at the 4-cell stage by activating PKC [67], but it is still unclear how the embryo controls the initial timing of compaction.

Compaction increases cell-to-cell adhesion and changes the cell shape. At the same time as compaction occurs, an asymmetric distribution of cellular components forms within each cell, which is referred to as polarization. Polarization occurs simultaneously with compaction at the 8-cell stage and is defined as a structurally and functionally asymmetric organization of cellular components. Even though polarization and compaction occur at the same time and place, they are two relatively independent processes, and compaction can occur in the absence of polarization [68] and vice versa [64]. Although the two processes are independent, they still influence each other. During polarization, the direction of polarization is always perpendicular to cell contact, which means the outside surface that has no contact with another cell surface will form the apical domain and the interior surface will form the basolateral domain. The apical domain is enriched in apical polarity proteins, such as the Par3-Par6-aPKC complex, and cytoskeletal components also accumulate in the region [69].

The molecular mechanism of polarization has become better understood in recent years, and several proteins have been found to participate in or affect the establishment of polarization. The asymmetric location of the Par3-Par6-aPKC complex is involved polarization. When the embryo starts polarization, this complex locates to the apical domain, whereas Par1 locates to the basolateral domain [70]. Lgl is also located on the basolateral domain, which maintains polarization through an antagonistic association with the Par3-Par6-aPKC complex [71]. Recently, phospholipase C (PLC)-mediated PIP2 hydrolysis has been shown to be a pre-requisite for the asymmetric location of the Par3-Par6-aPKC complex. PKC is activated by PLC hydrolyzing PIP2. Activated PKC activates RhoA leading to apical cortical accumulation of actomyosin, and ultimately actomyosin and the Par3-Par6-aPKC complex together establish the polarization of the embryo [72].

#### 2.2.2 The first cell fate decision

The establishment of polarization is very important for embryonic development. Before polarization, all three divisions are considered symmetric. However, for the next two divisions, cells can either undergo asymmetrical division that produces two polarized daughters or asymmetrical division that produces one polarized daughter and one non-polarized daughter. Non-polarized cells, which are on the inside of the blastomere, will differentiate into the ICM, while polarized cells on the outside of the blastomere will differentiate into the TE. The blastomere produces a constant number of ICM and TE cells by controlling the two divisions. Before polarization, all cells are totipotent. Upon differentiation the TE loses its totipotency; however, the ICM can differentiate into TE, which maintains totipotency.

An important question is why outer cells differentiate into TE while inner cells develop into the ICM. CDX2 is a key protein in TE specification, and *Cdx2* is expressed in all cells at the 8-cell stage. Upon polarization, however, *Cdx2* is expressed only in the TE at the blastocyst stage. CDX2 is a transcription factor that promotes cell differentiation into TE and represses ICM-specific gene expression at the 16 to 32-cell stage [73]. The expression of *Cdx2* is controlled by the transcription factors YAP1 and TEAD4. Unphosphorylated YAP1 can enter the nucleus and interact with TEAD4, leading to *Cdx2* transcription [74]. Furthermore, the Hippo signaling pathway, which is controlled by polarization, controls the phosphorylation of YAP1. Specifically, AMOT is the member of the Hippo signaling pathway that controls the activation of the pathway. After polarization, the location of AMOT in the inner and outer cells is different. In the outer cells, AMOT is located in the apical domain, whereas in the inner cells it is located in adherens junctions. AMOT interacts with E-cad via NF2, and this interaction allows LAT1/2 to phosphorylate AMOT. The phosphorylation of AMOT causes it to detach from the cortical F-actin, and it is stabilized in the adherens junctions where it forms a complex with LAT1/2. This complex activates the Hippo signaling pathway, and activation of the pathway ultimately leads to the phosphorylation of YAP1 [75,76]. In outer cells, AMOT does not interact with E-cad because it is limited to the apical domain, and this localization of AMOT means that the Hippo signaling pathway will not be activated. Besides controlling *Cdx2* expression, the Hippo signaling pathway might also be involved in the expression of *Sox2* to promote ICM development [77].

When inner and outer cells show differences in Hippo signaling pathway activation, their transcription patterns will be different. Outer cells, which develop into the TE, express specific TFs for TE maturation. In addition to CDX2 as described above, EOMES, and GATA3 are two TFs that activate TE marker genes such as *Hand1* and *Pl1* [78]. Inner cells express some TFs that are essential for pluripotency, like OCT4, NANOG, and SOX2. OCT4 is the key factor for inner cells to develop into the ICM [79], and OCT4 and CDX2 have a mutual inhibitory effect in the mouse embryo. A decrease in OCT4 in the ICM will lead to the expression of CDX2 and the loss of pluripotency [73].

The TE is an epithelial layer held together by tight junctions that wraps around the ICM. During TE establishment, tight junctions and DSM together help stabilize epithelial cohesion and integrity in the developing blastocyst [80]. At the 32-cell stage, the outer cells are fully committed to the TE lineage, and the embryo forms a fluid-filled cavity in its center called the blastocoele. The major force driving the accumulation of the fluid is the osmotic pressure. Na+/K+ pumps located on the basolateral side and Na+/H+ cotransporter on the apical side increase osmotic pressure via ion pumping, leading water to accumulate in the intercellular space. The pressurized fluid fractures cell-cell contacts into hundreds of micrometer-sized lumens. These micrometer-size lumens coarsen through a process akin to Ostwald ripening, and they ultimately converge into a single cavity giving the blastocyst its distinct morphology [81].

#### 2.2.3 The second cell fate decision

During the first cell fate decision, the embryo ultimately forms the blastocyst. Immediately afterwards, the ICM will face a second fate choice and the ICM will develop into the Epi, which will develop into the embryo, and the PrE, which will develop into extraembryonic tissues.

At the 32-cell stage, all ICM cells co-express marker TFs for the Epi and PrE like NANOG, OCT4, and GATA6. However, some ICM cells will downregulate *Nanog* and upregulate *Gata6* at the 64-cell stage [82]. This ultimately leads to the differential expression that will define the Epi and PrE lineages. The Epi specifically expresses *Nanog, Sox2,* and *Oct4*, while the PrE specifically expresses *Gata6, Pdgfra*, and *Sox17* [83]. This lineage differentiation is described by the “salt and pepper pattern”. At about the 64-cell stage, the ICM cells randomly adopt the Epi or PrE transcription pattern and the two types of cells mix like salt and pepper. Then at about the 100-cell stage these cells begin the separate, and the PrE cells will migrate until they reach the surface of the ICM exposed to the blastocoel [84].

NANOG and GATA6 inhibit each other's expression and undergo balanced expression in the ICM at the 32-cell stage [85]. However, differentiation of the ICM is considered to cause the unbalanced expression of *Nanog* and *Gata6*. FGF4 is a secretory protein that is considered to induce this unbalance, and FGF4 continues to be expressed during early embryonic development [86]. FGFR1/2, which is located on the surface of the ICM, receives the FGF4 signals and activates the ERK signaling pathway that promotes the expression of *Gata6* and thus disrupts the balance between NANOG and GATA6 [87].

The question is how this unbalance is established at the proper time. NANOG inhibits *Gata6* expression and promotes *Fgf4* expression, while GATA6 inhibits *Nanog* expression and promotes *Fgfr1/2* expression. FGF4, which activates ERK via FGFR1/2, promotes GATA6 expression. NANOG, GATA6, and FGF4-FGFR1/2-ERK coexist at steady states in ICM cells and show no differences in transcription pattern prior to the 64-cell stage [85]. Mutually exclusive expression of *Fgf4* and *Fgfr1/2* occurs earlier than unbalanced expressions of *Nanog* and *Gata6*. It seems that unbalanced expression of *Fgf4* and *Fgfr1/2* lead to unbalanced expression of *Nanog* and *Gata6*. Some studies suggest that small disturbances during development, for example, blastocyst cavity formation or transcriptional noise, might break the fragile equilibrium leading to different cell fates [88,89].

## 3 Pathogenesis of early embryonic arrest

### 3.1 Aneuploidy

Chromosomal abnormalities are a primary cause of preimplantation arrest of human embryos. Aneuploidy (an abnormal number of chromosomes) is the most common type of chromosome abnormality in humans with an incidence above 5% [90]. Most chromosomal abnormalities cannot be passed to the next generation because they commonly lead to early embryonic arrest, failed implantation, or miscarriage. Many events such as meiotic and mitotic defects, DNA damage, and fertilization errors can result in aneuploid embryos.

#### 3.1.1 Meiotic origin

Spermatocytes and primary oocytes form functional haploid gametes via meiosis I and meiosis II, wherein the DNA only replicates once to produce sister chromatids. During the pachytene stage of prophase I, non-sister homologous chromatids crossover, recombine, and exchange maternal and paternal chromosomal regions. Primary oocytes in neonatal ovaries are arrested at the diplotene stage of meiosis I until just before ovulation in the menstrual cycle. Upon progressing into metaphase I, homologous chromosomes align on the metaphase plate. Then microtubules will attach to kinetochores, separating the two homologous chromosomes to opposite poles. During meiosis II, sister chromatids are separated after degradation of cohesin via the separase protease and are pulled apart by opposing microtubules. Compared with spermatogenesis, the more complicated meiotic process of oogenesis leads to far more frequent maternal meiotic errors. The large number of IVF and ICSI cycles that have been performed have shown that the incidence of maternal meiotic errors is relatively high compared to other mammalian species, with approximately 20–25% of human oocytes carrying a chromosomal imbalance [91]. When fertilized, aneuploid oocytes will inevitably produce aneuploid embryos that will most likely fail to survive. However, the estimates of aneuploidy in mouse oocytes are typically only about 0.05–1% [92]. On the other hand, the frequency of aneuploidy in normal sperm samples is 6–7%, and this frequency significantly increases in cases of severe oligospermia or azoospermia due to testicular failure [93]. The incidence of aneuploidy in human oocytes drastically increases with maternal age, and up to 50% of clinically assessed pregnancies in women in their 40s are diagnosed as aneuploid [94].

Regardless of whether the errors occur in either metaphase I (MI) or MII oocytes, all aneuploidies of meiotic origin will pass onto the zygote. For instance, abnormal assembly, chiasmata formation, and recombination in spermatocytes and primary oocytes can lead to failed disjunction of homologous chromosomes in MI, thus generating univalents that segregate randomly to the daughter cells. Rare deleterious alterations in genes involved in chromosome recombination in meiosis, such as *SYCP3, HFM1, SYCE1, PSMC3IP*, and *SGO2*, will cause meiotic disorders, aneuploidy, embryonic arrest, recurrent miscarriage, human infertility, etc [95].

The spindle assembly checkpoint (SAC) is a monitoring mechanism for meiosis and mitosis that prevents the missegregation of sister chromatids. The SAC consists of MPS1, Aurora, and several proteins from the MAD and BUB families, and it prevents CDC20 from activating the anaphase-promoting complex/cyclosome (APC/C) prior to the congregation and orientation of all chromosomes on the metaphase plate. APC/C^CDC20^ mediates the degradation of cyclin B1, thus rapidly inactivating CDK1 and allowing the cell to exit meiosis or mitosis. The mechanism of the male cell cycle checkpoint appears to be more powerful, successfully eliminating cells with meiotic errors regardless of paternal age. On the contrary, female SAC-mediated control of meiosis seems to be weaker, and it is activated only when several chromosomes are misaligned, and a single chromosome that fails to align properly can evade the SAC [96]. Therefore, oocytes are more prone to aneuploidy than sperm. Moreover, the ability to maintain SAC signaling decreases with maternal age because the expression levels of *MAD2* and *BUB1* are significantly reduced in older women, and this accelerates the first meiotic division due to premature APC^CDC20^ activity and increases the rate of aneuploidy [97].

The meiosis-specific cohesin complex consists of two structural proteins (SMC1β and SMC3, which are encoded by *SMC1B* and *SMC3*, respectively), an α-kleisin protein (RAD21, RAD21L, or REC8), and the stromal antigen protein STAG3, which together form a circular structure that holds sister chromatids together during meiosis and prevents their premature separation [98]. At the onset of anaphase, the APC/C^CDC20^ mediates a rise in separase activity that degrades the kleisin subunit REC8, thus unlocking the sister chromatids. Pathogenic variants in *REC8, SMC1B*, and *STAG3* are associated with primary ovarian insufficiency and human infertility [99].

In aged human and mouse oocytes, the expression level of cohesin is significantly reduced, making it impossible to stabilize chiasmata and bound sister chromatids and predisposing oocytes to the generation of univalents in MI and subsequent segregation errors [100]. The loss of cohesin with increasing female age results from the depletion of SGO2 that protects REC8 from cleavage [101]. Complete loss of cohesin along the whole chromosome with age is an extreme event, which will result in independent segregation of sister chromatids during MI. The less severe age-related loss of cohesin may occur on the centromere or chromosome arm. The loss of cohesin around the centromere leads to an increase in the distance between kinetochores of sister chromatids with increasing female age, predisposing sister kinetochore pairs to forming bipolar attachments to microtubules thus making the homologous chromosomes prone to missegregation. Additionally, the age-associated loss of cohesin on the chromosome arms might lead to bivalents with distal exchange events in MI oocytes, thus making them more susceptible to undergoing missegregation.

#### 3.1.2 Fertilization errors

In human zygotes, the paternal centrosome nucleates microtubules to form the sperm aster, which allows the female pronucleus to bind to microtubules and move towards the male pronucleus in a dynein-dependent manner. During pronuclear fusion in zygotes, the sperm centrosome duplicates and separates to generate the mitotic spindle, which pulls the chromosomes apart in the first post-zygotic division. However, the first cleavage does not occur as expected in some fertilized oocytes. The time-lapse analysis of human early embryos in vitro showed that zygotes with three pronuclei (3PN) are highly prone to the tripolar division that carries either an extra set of haploid paternal or maternal chromosomes, while 2PN embryos also displayed defective first cleavages [102]. When mitotic mosaicism is detected in embryos during IVF attempts, this may be due to defects in the sperm used for fertilization. The presence of two sperm in human zygotes leads to 3PN and mosaic embryos, and the abnormal sperm centrosome induces mitotic mosaicism in embryos.

In addition, absent or genetically aberrant paternal RNAs and proteins may also cause genomic instability in early embryos. A sperm actually contains up to several thousand different RNAs that retain both coding and specific non-coding RNAs, some of which have known roles in mitotic progression and in preventing aneuploidy. One of these microRNAs, miR-34c, is inherited from sperm but is detected in zygotes. The first cleavage becomes significantly suppressed after blocking paternal miR-34c in mouse zygotes [103]. Moreover, it has been proposed that a small subset of paternal transcripts inherited from human sperm is significant for maintaining early embryogenesis from the 3 to 4-cell stage of human preimplantation embryos [104,105]. The presence of functional sperm RNAs suggests that male factors also contribute to the transition from oocyte to embryo. In addition, the persistence and integrity of sperm RNAs may be affected by aging or by environmental factors.

#### 3.1.3 Mitosis origin

As many as 80% of chromosomal imbalances in human embryos result from mitotic defects and cause embryonic mosaicism [106]. Compared to meiotic errors, mitotically derived mosaic aneuploidies in embryos are generally not correlated with advanced maternal age. Accurate cleavage divisions also rely on the intact assembly of the mitotic spindle and the integrity of the SAC to ensure proper separation and segregation of chromosomes to daughter cells. As embryonic cleavage proceeds, mitotic errors, including excessive centrosome duplication, loss of cohesion, weaker SAC, impaired spindle assembly, and cytokinesis can contribute to the missegregation of chromosomes and to aneuploid embryos. For example, the polo-like kinase 4 (PLK4) plays a central role in parental centriole duplication by recruiting centriole biogenesis proteins such as SASS6, CENPJ, CCP110, CEP135, and gamma-tubulin. A deleterious variant in *PLK4* was shown to cause aneuploidy by disrupting the centrosome cycle, and it occurred at a higher frequency in women who generated aneuploid embryos only due to mitotic errors [107]. Similarly, CEP57 regulates the pericentriolar material (PCM) organization and centriole engagement. Therefore, depletion of CEP57 causes PCM disorganization and premature centriole disengagement in mitosis, resulting in chromosome missegregation, and homozygous pathogenic variants in *CEP57* were identified in patients with mosaic variegated aneuploidy syndrome [108]. Studies in mouse embryos have shown that the knockdown of the spindle assembly complex components, such as BUB3, BUBR1, and MAD2, results in inappropriate chromosome segregation, aneuploidy, and micronuclei [109]. In human embryos, the *BUB1B* gene encodes a mitotic checkpoint serine/threonine kinase B that plays an important role in the regulation of the SAC, and rare bi-allelic variants in *BUB1B* were identified in fetuses affected by mosaic aneuploidy [110].

Regardless of the meiotic or mitotic origin, the two main mechanisms causing chromosome missegregation are non-disjunction and anaphase lagging, both of which contribute to aneuploid embryos and embryonic arrest. During non-disjunction, the sister chromatids fail to separate at the centromere during the metaphase stage, and this results in the loss of a chromosome in one daughter cell and a corresponding additional chromosome in the other daughter cell. Thus non-disjunction that occurs during meiosis or mitosis will produce an aneuploid zygote or embryonic mosaicism, respectively. Anaphase lagging occurs when a chromatid cannot connect or detaches prematurely from the spindle and is thus left behind during cytokinesis, generating a diploid daughter cell and one cell with monosomy during meiosis or mitosis. In addition, chromosome lagging commonly appears due to the formation of erroneous merotelic kinetochore-spindle microtubule attachments, whereby the kinetochore on a single chromatid is simultaneously attached to microtubules emerging from opposite spindle poles. Subsequently, the lagging chromosomes can form micronuclei in the cytoplasm and undergo defective DNA replication, which produces DNA damage and double-strand breaks (DSBs). Such DSBs within micronuclei can trigger an error-prone non-homologous end-joining repair mechanism, resulting in chromosomal aberrations and chromosomal fragments that provide the basis for cellular fragmentation in human embryos.

Human preimplantation embryos express enhanced cell cycle drivers and reduced cell cycle checkpoints that might make blastomeres more susceptible to anaphase lagging during early mitotic divisions. The cell cycle control becomes apparent only after ZGA in human embryos [111]. Therefore, permissive cell cycle checkpoints might allow aneuploid embryos to proceed through mitosis even if the chromosomes have not been properly aligned on the mitotic spindle at the metaphase stage. Conversely, compared to other cell types the embryo enters anaphase with highly expressed cell cycle drivers and shortened cell division intervals, thus losing the lagging chromosomes when cytokinesis occurs.

#### 3.1.4 DNA damage

The DNA damage response and repair (DDR) pathway involves cell cycle checkpoint activation and DNA damage repair and tolerance mechanisms. ATM and ATR are the most upstream kinases of the DDR pathway, and these activate the cell cycle checkpoint protein CHK1 that regulates mitosis through several mechanisms [112]. The DDR pathway is more vulnerable to chromosome segregation errors in preimplantation embryos because the DNA damage checkpoint and repair might be insufficient in embryos prior to ZGA possibly due to limited ATM kinase activity [113]. In addition, the kinase ATM phosphorylates histone H2AX at the site of DSBs, which recruits the necessary DNA repair proteins [114]. Thus, the loss of ATM activity in early embryos compromises the phosphorylation of key downstream targets involving DSB repair, consequently resulting in genomic instability [115]. Therefore, the early embryos that lack proper ATM activity are error prone during mitotic divisions.

Functional apoptotic pathways seem to be suppressed in early human embryos upon massive DNA damage, thus facilitating the survival of the embryos with damaged DNA or aberrant chromosomes until later stages [116]. Some essential G1 and G2 cell checkpoint proteins, such as RB and WEE1, are silenced in cleavage embryos [117], and SAC components, like BUB1, BUB3, and PTTG1, and the cell cycle regulator TP53 are differentially expressed in human aneuploid embryos compared to euploid embryos [118]. Such events lead to more permissive embryonic cell cycle checkpoints that allow for rapid cleavage divisions. Human oocytes can be surprisingly inefficient in responding to DNA damage, thus displaying poor competence to maintain genomic stability in early embryos prior to ZGA. Thus, blastomeres with damaged or incompletely replicated DNA can still undergo mitosis, thus bypassing SAC-mediated arrest and continuing to progress through cell division, which leads to increased chromosomal aberrations and aneuploidy in cleavage-stage embryos.

### 3.2 Genetic causes of early embryonic arrest

Early embryonic arrest is one of the main causes of human infertility, but it is difficult to diagnose because a preimplantation embryo might undergo arrest even before a woman notices her pregnancy in cases of in vivo human conceptions. The genetic causes of early embryonic arrest are maternal or paternal in origin, but they are largely unknown and are difficult to identify. Moreover, both maternal and paternal factors can contribute together to cause early embryonic lethality, as suggested by hundreds of knockout animal models.

#### 3.2.1 Pathogenic variants of maternal origin

Some defects that occur in the processes of human oocyte maturation will lead to preimplantation embryonic lethality and female infertility. In recent years, a few genes (including *TUBB8, PATL2, BTG4, TRIP13, REC114, MEI1, ASTL, FBXO43, TLE6, PADI6, CDC20, NLRP2, NLRP5*, and *KHDC3L*) involved in oocyte maturation have been shown to be responsible for a spectrum of early embryonic lethality phenotypes in humans, including oocyte maturation arrest, fertilization failure, early embryonic arrest, and embryonic implantation failure (Table 1) [119]. These pathogenic variants in the above genes have two major patterns of Mendelian inheritance, including autosomal dominant and recessive inheritance.

**Table 1:** **The genetic causes of human early embryonic arrest**.

Gene	Gene MIM number	Mode of inheritance	Phenotype in mutants	Phenotype MIM number	Refs
*PADI6*	610363	AR	Early embryonic arrest	617234	[120]
*TLE6*	612399	AR	Fertilization failure	616814	[[121], [122]]
			Early embryonic arrest		
			Embryonic implantation failure		
*NLRP2*	609364	AR	Early embryonic arrest	/	[123]
*NLRP5*	609658	AR	Early embryonic arrest	/	[123]
			Fertilization failure		
*KHDC3L*	611687	AR	Early embryonic arrest	614293	[124]
			Recurrent hydatidiform moles		
*TUBB8*	616768	AD, AR, de novo	Oocyte maturation arrest	616780	[[125], [126]]
			Fertilization failure		
			Early embryonic arrest		
			Embryonic implantation failure		
*PATL2*	614661	AR	Oocyte maturation arrest	617743	[127]
			Fertilization failure		
			Early embryonic arrest		
*BTG4*	605673	AR	Zygotic cleavage failure	619009	[10]
*TRIP13*	604507	AR	Oocyte maturation arrest	619011	[128]
			Zygotic cleavage failure		
*REC114*	618421	AR	Multiple pronuclei formation	619176	[129]
			Early embryonic arrest		
			Recurrent hydatidiform moles		
			Miscarriage		
*MEI1*	608797	AR	Azoospermia	618431	[130]
			Early embryonic arrest		
			Recurrent implantation failure		
			Recurrent hydatidiform moles		
			Recurrent pregnancy loss		
*ASTL*	608860	AR	Poor fertilization	619643	[131]
			Early embryonic arrest		
*FBXO43*	609110	AR	Early embryonic arrest	619697	[132]
*CDC20*	603618	AR	Oocyte maturation arrest	/	[133]
			Fertilization failure		
			Early embryonic arrest		
*ACTL7A*	604303	AR	Poor fertilization	/	[134]
			Early embryonic arrest		

AD, autosomal dominant; AR, autosomal recessive.

The SCMC complex consists of PADI6, TLE6, NLRP2, NLRP5, KHDC3L, and OOEP and is essential for embryonic activation and development. *PADI6* was identified as a causative gene in patients with early embryonic arrest observed in failed IVF/ICSI attempts [120]. Bi-allelic variants lead to the lack of PADI6 protein in patients’ oocytes, reducing the expression levels of a few genes that regulate ZGA in their embryos, which results in early embryonic arrest. Maternal homozygous or compound heterozygous variants in *TLE6* are responsible for fertilization failure, early embryonic arrest, and embryonic implantation failure [121]. The variants impair the expression and phosphorylation of TLE6, thus preventing the interaction between TLE6 and other members of the SCMC (KHDC3L and OOEP) [122]. Moreover, bi-allelic variants in *NLRP2* and *NLRP5* were found to be responsible for fertilization failure or early embryonic arrest in humans [123]. There is phenotypic variability in patients with *NLRP2* variants, and different *NLRP2* variants influence protein function to different extents. It is worth noting that some patients with bi-allelic missense variants in *NLRP2* are able to obtain a few viable embryos and can eventually give birth to infants after several IVF/ICSI attempts. In addition to recurrent hydatidiform mole, bi-allelic variants in *KHDC3L* were also identified to cause embryonic arrest at the cleavage or morula stage [124]. These studies provide further evidence that the SCMC in humans plays a critical role in early embryonic development.

TUBB8 is a primate-specific β-tubulin isotype specifically expressed in human oocytes and early embryos, and it is involved in the assembly of the oocyte meiotic spindle. Most *TUBB8* variants are either paternally inherited or occur de novo, and they follow a dominant inheritance pattern [125]. These heterozygous variants result in unfolded chaperones, impaired microtubule dynamics, and disorganized spindles due to the dominant-negative effect. On the other hand, homozygous loss-of-function variants are able to disrupt microtubule function and spindle assembly through insufficient haploid dose [126]. Different *TUBB8* variants lead to a variety of reproductive phenotypes, including oocyte maturation disorders, fertilization failure, early embryonic arrest, and embryonic implantation failure, ultimately resulting in female infertility. The RNA-binding protein PATL2 is a translational repressor and is highly expressed in human oocytes. *PATL2* variants may influence the expression of PATL2 proteins, further hindering canonical translational repression and protein synthesis [127]. Patients with bi-allelic *PATL2* variants exhibit phenotypic variability due to the variants’ different effects on PATL2 function. The oocytes or embryos of patients with greater impairment of PATL2 protein tend to arrest at earlier stages.

Zygotic cleavage failure is a unique early embryonic phenotype, and homozygous variants in *BTG4* were identified in patients with the disease, following a Mendelian recessive inheritance pattern [10]. BTG4 acts as a prime adaptor of the CCR4-NOT deadenylase complex, facilitating the decay of maternal mRNAs in early embryonic development. The *BTG4* variants either reduce the protein abundance of BTG4 or impair the interaction between BTG4 and the catalytic subunit of the CCR4-NOT complex, further disrupting the process of maternal mRNA decay that plays a key role in ZGA. During meiosis I, TRIP13 acts as a negative regulator of the cell cycle checkpoint proteins HORMAD1 and HORMAD2 and mainly participates in the recombination of homologous chromosomes [128]. TRIP13 opens the checkpoint by mediating the removal of HORMAD2 from synapsed chromosomal axes, thus enabling homologous chromosomes to be successfully separated. Bi-allelic missense variants in *TRIP13* reduce the protein level of TRIP13 and cause the accumulation of HORMAD2, thus hindering the separation of homologous chromosomes and ultimately leading to MI arrest or zygotic cleavage failure.

The assembly of the heterotrimeric complex IHO1-REC114-MEI4 at sites of DSBs is essential for the formation of DSBs that induce meiotic recombinational events. Homozygous splicing and missense variants in *REC114* result in a decrease in the protein level of REC114 and a loss of its ability to maintain the stability of MEI4, thus impairing the function of the IHO1-REC114-MEI4 complex [129]. Furthermore, the reduced protein level of IHO1-REC114-MEI4 may lead to incorrect localization of SPO11 that initiates DSBs and homologous chromosome recombination. Abnormal recombinational events further induce missegregation of chromosomes, resulting in defective oocyte maturation, as manifested by multiple pronuclei formation, early embryonic arrest, recurrent hydatidiform moles, and miscarriage [129]. The phenotypic variability may depend on the different effects of the variants. *MEI1*, which encodes the meiotic DSB formation protein 1, is required for the formation of genetically programmed DSBs that initiate meiosis. Several studies have shown that bi-allelic variants in *MEI1* cause azoospermia, recurrent hydatidiform moles, early embryonic arrest, recurrent implantation failure, and recurrent pregnancy loss [130].

Following fertilization, the hydrolases in the cortical granules of oocytes are released into the perivitelline space through exocytosis. One of these hydrolases, ovastacin, which is encoded by the *ASTL* gene, catalyzes the cleavage of the ZP2 protein that binds sperm on the surface of the zona pellucida, thus preventing further sperm from recognizing the oocyte. Moreover, ovastacin triggers the zona pellucida hardening that prevents other sperm from passing through the zona pellucida. Pathogenic variants affect the expression of *ASTL*, leading to the complete loss of ovastacin [131]. The oocytes retrieved from patients with *ASTL* variants are still able to block polyspermy to some extent and can produce viable 8-cell embryos. However, the lack of *ASTL* affects oocyte cytoplasmic maturation, resulting in poor fertilization and early embryonic arrest, ultimately leading to female infertility.

The MII arrest in oocytes is enforced by the cytostatic factor that maintains the stability of the M-phase promoting factor (MPF) by inhibiting APC/C activity. FBXO43 is an inhibitor of the APC/C and is a key component of the cytostatic factor. Fertilization leads to calcium-activated CAMKII that triggers the inactivation and degradation of FBXO43, eventually activating APC/C and resuming meiosis II in oocytes. Homozygous variants reduce the protein level of FBXO43 and impair the ability of FBXO43 to stabilize the downstream MPF component cyclin B1, resulting in meiotic errors that affect early embryonic development after fertilization [132]. CDC20 acts as a co-activator of APC/C. There is a low expression of *CDC20* in oocytes at the diplotene stage of meiosis I, but a high expression in human MII oocytes. Patients harboring homozygous or compound heterozygous variants in *CDC20* exhibit a phenotype of MI arrest or early embryonic arrest depending on the degree of CDC20 impairment resulting from different variants in *CDC20* [133].

#### 3.2.2 Pathogenic variant of paternal origin

There are a few genetic factors derived from sperm that have been shown to affect early embryonic development (Table 1). It was reported that a homozygous missense variant in *ACTL7A* was identified in two infertile brothers with normal semen parameters but presenting with poor fertilization and early embryonic arrest in IVF/ICSI cycles [134]. ACTL7A, located in the acrosome and tail of mature sperm, is a member of the actin-related protein family and plays an important role in spermiogenesis. This variant is deleterious and causes sperm acrosomal defects, leading to reduced expression and abnormal localization of PLCζ, which initiates calcium oscillations associated with oocyte activation, and thus is a potential cause of fertilization failure and early embryonic arrest [134].

#### 3.2.3 Homozygous deletion of genes associated with early embryonic lethality

**Fig. 3:**
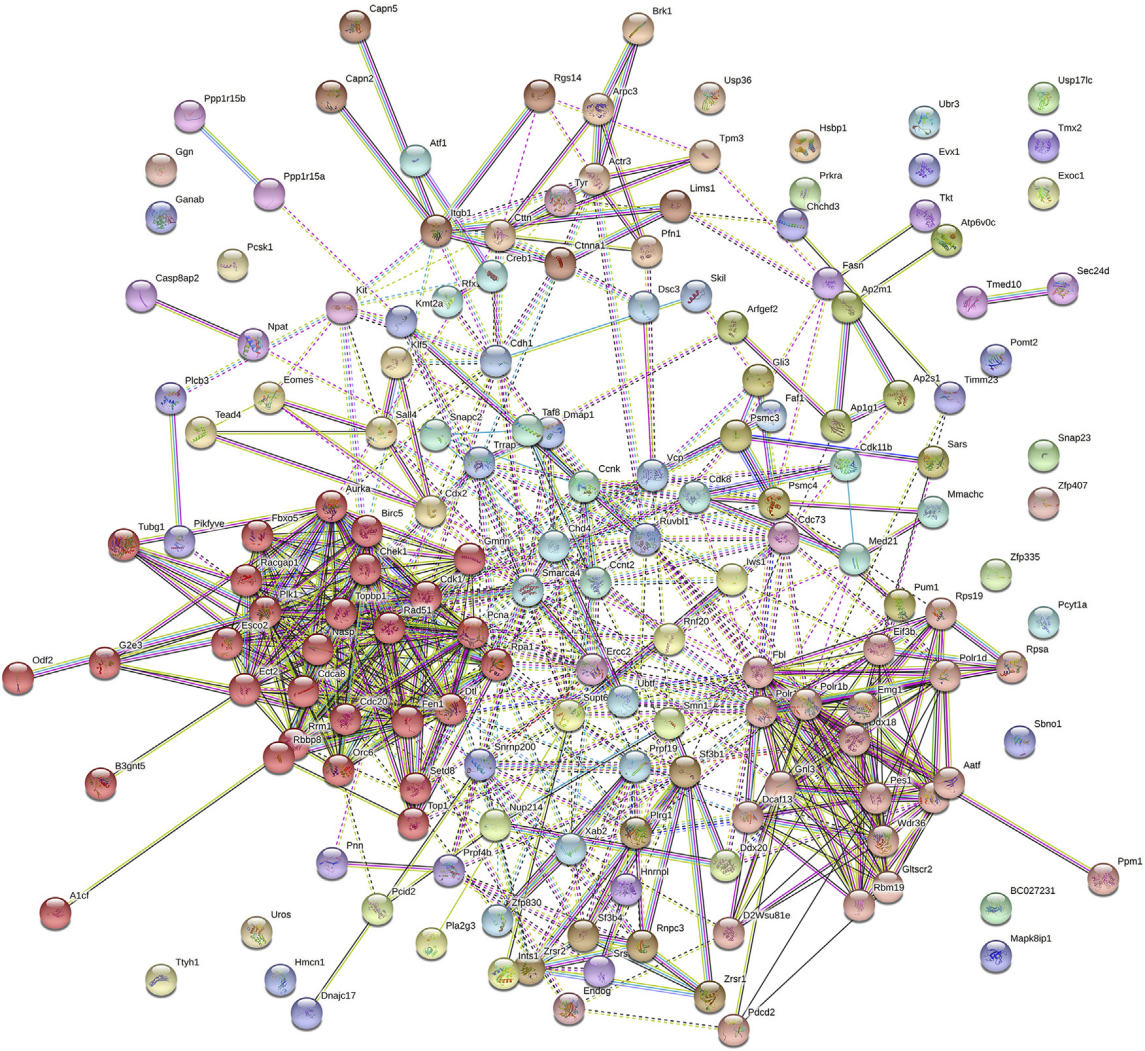
**The PPI network of the genes responsible for early embryonic lethality**. There are 162 genes involved in mouse knockout models that exhibit embryonic lethality prior to implantation with complete penetrance according to the Mouse Genome Informatics database. The PPI network of these genes was constructed from the STRING database, displaying an enrichment of genes regulating the cell cycle.

Hundreds of mouse knockout models have been shown to exhibit embryonic lethality before implantation with complete penetrance according to the Mouse Genome Informatics database. The gene ontology (GO) enrichment analysis of the genes identified in these models described above was performed by DAVID (Table 2). The significantly enriched biological processes focus primarily on blastocyst development, cell cycle, mRNA processing, and cell division. Moreover, a protein-protein interaction (PPI) network of these genes was constructed from the STRING database (Fig. 3). The MCL clustering algorithm was applied to analyze the PPI network. With a threshold of inflation parameter > 3, a cluster with 29 candidate hub genes was highlighted.

**Table 2:** **GO analysis of the genes responsible for embryonic lethality before implantation with complete penetrance in knockout mice**.

Term of biological process	Count	%	Benjamini P-Value	-Log_10_ (FDR)
blastocyst development	11	6.8	1.90E-10	9.72
cell cycle	26	16	5.60E-08	7.25
mRNA processing	18	11.1	2.30E-06	5.64
RNA splicing	16	9.9	2.30E-06	5.64
cell division	18	11.1	6.10E-06	5.21
in utero embryonic development	16	9.9	3.30E-05	4.48
DNA repair	16	9.9	8.00E-05	4.10
mRNA splicing, via spliceosome	11	6.8	2.50E-04	3.60
inner cell mass cell proliferation	5	3.1	1.70E-03	2.77
cellular response to DNA damage stimulus	16	9.9	1.80E-03	2.74
mitotic G2 DNA damage checkpoint	6	3.7	1.80E-03	2.74
DNA replication	8	4.9	6.70E-03	2.17
mitotic cell cycle	8	4.9	8.40E-03	2.08
regulation of cell cycle	11	6.8	1.60E-02	1.80
embryo implantation	6	3.7	1.60E-02	1.80
macromolecular complex assembly	7	4.3	3.10E-02	1.51
stem cell population maintenance	6	3.7	3.10E-02	1.51
regulation of DNA replication	5	3.1	3.50E-02	1.46
regulation of mitotic cell cycle	5	3.1	4.30E-02	1.37
nucleolus organization	3	1.9	4.30E-02	1.37

Some hub genes, such as *Fbxo5, Racgap1, Aurka, Birc5, Cdc20, Cdca8, Cdk1, Ect2, Plk1*, and *Rbbp8*, are also key enriched genes in the biological processes (cell cycle and cell division) of GO enrichment analysis. For instance, Fbxo5 is a mitotic regulator that interacts with Cdc20 and inhibits APC/C activity. Racgap1 plays a regulatory role in cytokinesis, cell growth, and differentiation. Aurka regulates microtubule formation and stabilization at the spindle pole during chromosome segregation. Birc5 and Cdca8 are the components of a chromosome passage protein complex which is significant for chromosome alignment and segregation during cell division. Cdk1 is a catalytic subunit of the MPF that is essential for G1/S and G2/M phase transitions of cell cycle. Ect2 catalyzes guanine nucleotide exchange on the Rho family members specifically and involves the regulation of cytokinesis. Plk1 is a Ser/Thr protein kinase that performs several important functions throughout M phase of the cell cycle. Rbbp8 regulates Chek1 activation and controls cell cycle checkpoint. Thus, a large number of genes involved in mitosis are essential for early embryonic development.

### 3.3 Mitochondrial defects result in early embryonic arrest

Mitochondria are at the heart of intracellular redox metabolism because they produce reactive oxygen species (ROS, the universal source of intracellular oxidative stress) and generate the TCA cycle intermediates and reducing equivalents (NADH and NADPH) needed for antioxidant defense. Therefore, any change in mitochondrial function in the embryo will be reflected in changes in the intracellular redox state. Many pathological conditions or environmental insults impair early development by altering mitochondrial function. For instance, maternal diabetes can expose embryos to high glucose levels that cause the downregulation of glucose transporters and mitochondrial defects that are reflected in reduced TCA cycle activity and reduced cellular ATP levels in oocytes [135]. Aging may affect mitochondrial integrity in oocytes, possibly through cumulative oxidative damage produced by ROS, consequently impairing the mitochondria's ability to produce ATP. The potential accumulation of maternal mitochondrial DNA (mtDNA) variants can also lead to mitochondrial defects in the years between fetal oogenesis and meiotic resumption, and oocytes from aging women have been shown to exhibit increased mtDNA variability and decreased ATP production [136]. Because ATP production is required for microtubule movement and cytoskeletal actin filament contraction during meiosis and mitosis, the separation of chromosomes by meiotic spindle is highly energy dependent. The pathological conditions, including maternal diabetes, aging, and maternal mtDNA variants, might indirectly affect chromosome segregation and cell division.

Oxidants may be produced endogenously by intracellular stress signaling that involves mitochondrial ROS production. The production of mitochondrial ROS increases dramatically in mammalian blastocysts [137], and the antioxidant defenses of the embryo (especially the mitochondria) must cope with this increased endogenous oxidant load in order to maintain development. The oocytes maintain the antioxidant defenses in early embryos in order to respond to exogenous and endogenous oxidant damage that occurs in early development. For example, mitochondria provide ATP for the production of glutathione during oocyte maturation and also participate in the regeneration of NADPH and glutathione during early development, which serves to rejuvenate antioxidant defenses [138]. Mammalian oocytes and embryos are likely to experience a slow but continuous ROS buildup during development, but this ROS production causes oxidative stress only when the embryo's antioxidant defense system is overwhelmed. Severe oxidative stress can lead to breakdown of DNA, lipids, and proteins and in extreme cases can cause apoptosis or necrosis [139].

Telomere attrition plays a central role in oocyte senescence. Oocytes ovulated by elderly women exit from the oogonial cell cycle later during fetal life, followed by cumulative exposure to ROS due to long-term stagnation in the adult ovaries resulting in shorter telomeres in elderly women's oocytes. Spermatogonia can evade telomere attrition by expressing telomerase throughout the life of a man, and telomerase activity in oocytes and cleavage embryos is low to absent [140]. Telomere attrition in oocytes and cleavage embryos produces anaphase lagging, chromosome bridges, micronuclei, and genomic instability through non-homologous end joining, which results in aneuploidy, mosaicism, and copy number variations by chromothripsis and alternative lengthening of telomeres (ALT) [141]. ALT is a recombination-based telomere elongation mechanism that facilitates telomere elongation in preimplantation embryos and involves extensive telomere sister chromatid exchange and nuclear localization of proteins that mediate DSB repair [142]. ALT robustly rebuilds telomeres, but also tends to cause genomic instability.

Pre-implantation development in mammals is very sensitive to oxidative stress, which can induce embryonic stagnation at ZGA by inactivating the CDC25 phosphatase [143]. CDC25 can activate MPF to allow entry into mitosis, and it is inactivated by oxidation of two cysteines in its catalytic site. Embryos are blocked at the G2/M transition of the cell cycle when they accumulate pre-MPF that has never been activated by dephosphorylation. After ZGA, the apoptotic cascade is turned on and oxidative stress tends to induce apoptosis rather than blocking the cell cycle. Arrested embryos contain more p66shc than healthy embryos [144]. p66shc is an adaptor that binds to cytochrome c oxidase in mitochondria and increases the production of mitochondrial ROS [145]. The enhanced mitochondrial ROS production induced by p66shc initiates the apoptotic cascade when the embryos are unable to restore the reducing intracellular redox state.

### 3.4 Other causes of early embryonic arrest

The effect of genetic material on embryonic development is obvious, but maternal physiology, psychology, and behaviors can also affect embryonic development. For example, smoking increases the risk of embryonic development arrest. Thus, the effect of extrinsic factors on embryonic development should not be ignored.

In mammals, the oviduct is where fertilization and early embryonic development take place. The oviduct is filled with oviductal fluid that is secreted from the oviduct's epithelial cells. Oviductal fluid contains metabolites, amino acids, proteins, lipids, and inorganic salts and thus provides the various nutrients needed for embryonic development. OVGP1, also known as oviductin, is the major non-serum glycoprotein present in the oviductal fluid. Early embryonic development can be arrested at the 2-cell stage by antibodies against a C-terminal peptide of OVGP1 in mice, but *Ovgp1*-null mice reproduce normally [146]. The function of *OVGP1* in mammalian reproduction thus needs further research.

Studies have suggested that psychological stress is involved in a series of biological events that act on the reproductive system, causing early embryonic development arrest [147]. Psychological stress directly influences hormone signals in the body, and the hypothalamic-pituitary-adrenal axis responds to stress and has effects on embryonic development. When psychological stress occurs, the hypothalamic-pituitary-adrenal axis can stimulate the pituitary to secrete an adrenocorticotropic hormone that subsequently leads to glucocorticoid secretion. Increasing glucocorticoid impairs embryonic development through the Fas system [147]. Psychological stress induces oxidative stress, and even though ROS are beneficial for embryonic development the overaccumulation of ROS can result in oxidative stress. Oxidative stress negatively affects embryonic development by increasing DNA damage, destroying proteins, and triggering apoptosis [148].

A mother's behaviors or habits can influence the embryo, and some poor behaviors might result in embryonic development arrest. There is a consensus that smoking is harmful to health, but whether smoking affects embryonic development is not yet clear. Cigarette smoke contains thousands of metabolites, some of which are carcinogenic and genotoxic, including polycyclic aromatic hydrocarbons (e.g., benzo(a)pyrene), heavy metals (e.g., cadmium, lead, cobalt), alkaloids (e.g., nicotine), etc., all of which might have toxicities in embryonic development. Epidemiological and clinical statistical studies on different populations show that smoking has no significant effect on embryos compared with not smoking but does decrease the implantation rate in IVF attempts [149]. Thus the effect of smoking on human embryonic development requires further study. Although it is inconclusive in humans, animal models suggest that cigarette smoke affects embryonic development, and mice exposed to cigarette smoke show increased egg fragmentation, delayed fertilization, and decreasing cleavage [149]. Cadmium is an abundant toxic metabolite of cigarettes, and cadmium can be detected in female follicular fluid and male seminal plasma in those who smoke. Wild-type embryos exposed to 5 µM cadmium also exhibit reduced blastocyst development, reduced telomere length, and decreased numbers of OCT4-positive cells in the embryo [150]. Benzo(a)pyrene is another metabolite that may be harmful to the embryo, and benzo(a)pyrene exposure and decreasing numbers of OCT4 and NANOG-positive cells might affect embryo differentiation into the Epi and PrE lineages in mice [151].

## 4 Conclusion and perspective

The embryo develops from a single cell into a multicellular blastocyst, and this developmental process is guided by complex and delicate regulatory mechanisms. Benefiting from in vitro embryo culture technology, the morphological changes in early embryonic development have been clearly described. With the advancement of molecular biology techniques, research into the molecular mechanism of early embryonic development has become more and more in-depth. Widespread use of animal models and sequencing technology has given us a clearer understanding of early embryonic development and of the pathological mechanisms of embryonic development. Although much has been learned about early embryonic development, there are still many unanswered questions, such as how embryos maintain relatively stable ICM cell numbers. Physiological and pathological research on embryonic development is therefore still an important subject for future studies.

## Declaration of competing interest

The authors declare that they have no conflicts of interest in this work.
